# Supplementary material for: In Vitro Cultures of Scutellaria brevibracteata subsp. subvelutina as a Source of Bioactive Phenolic Metabolites
Source: Molecules. 2023 Feb 14;28(4):1785. doi: 10.3390/molecules28041785 (PMC9964101; doi:10.3390/molecules28041785)

Figure S1. The brine shrimp (*Artemia salina*) surviving larvae (%) after 24 h of incubation with *S. brevibracteata* subsp. *subvelutina* extract

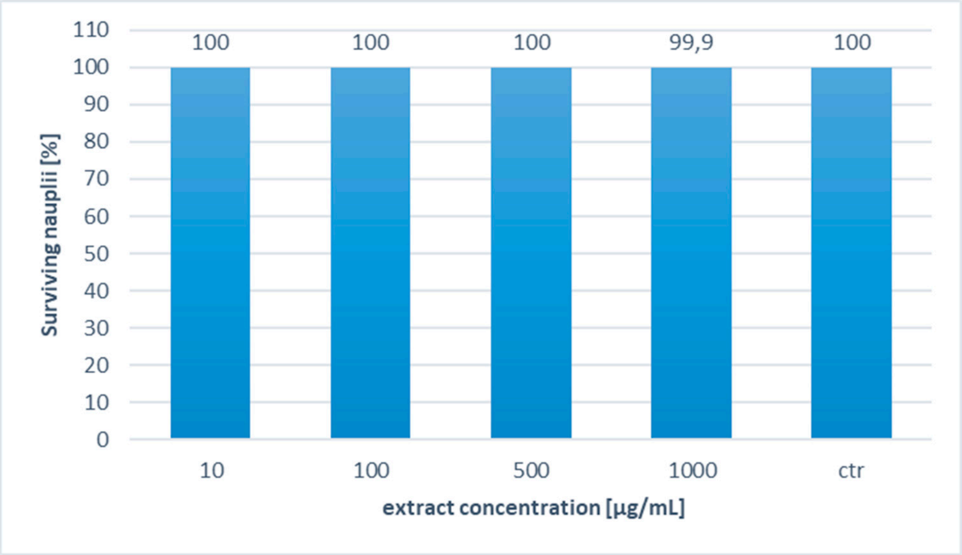

Supplement: Supplementary file 1 [file molecules-28-01785-s001.zip › molecules-2221539-supplementary.pdf]
